# 滇东产(燃)煤区农民肺癌流行病学调查

**DOI:** 10.3779/j.issn.1009-3419.2011.02.02

**Published:** 2011-02-20

**Authors:** 继华 李, 云生 张, 云 李, 国青 殷, 玥冰 李, 伯福 宁, 家敏 国

**Affiliations:** 1 655000 曲靖，曲靖市疾病预防控制中心 Qujing Centers for Disease Control and Prevention, Qujing 655000, China; 2 100048 北京，首都师范大学 Te Capital Normal University, Beijing 100048, China; 3 655400 宣威，宣威市疾病预防控制中心 Xuanwei Centers for Disease Control and Prevention, Xuanwei 655400, China; 4 655500 富源，富源县疾病预防控制中心 Fuyuan Centers for Disease Control and Prevention, Fuyuan 655500, China

**Keywords:** 肺肿瘤, 筛查, 流行病学, 烟煤, 滇东地区, Lung neoplasms, Mass screening, Epidemiology, Coal, Diandong area

## Abstract

**背景与目的:**

云南省宣威市位于滇东黔西晚二叠纪聚煤区，当地肺癌死亡率居全国首位，研究显示宣威农民肺癌高发与室内烟煤燃烧产生的多环芳烃等污染物有关。近年来宣威周边产煤地区环境污染和肺癌高发问题引起国内外关注，滇东其它地区居民肺癌流行水平与发病原因是否与宣威相似未见报道。本研究旨在探索滇东产（燃）煤地区农民肺癌流行状况和发病原因。

**方法:**

采用多阶段、分层、整群、概率抽样方法随机抽取滇东产（燃）煤382个调查小区，对其中30岁-79岁常住居民进行X线胸片肺癌筛查和相关因素调查，筛查阳性疑似肺癌进行CT复查，诊断为肺癌的病例进行痰癌细胞检查和随访。采用世界肿瘤研究中心标化率比对不同地区肺癌筛查阳性率进行显著性检验分析、调整合并为A区、B区、C区、D区等4层，并进行肺癌相关因素分析。

**结果:**

共筛查和问卷调查52, 833名农村居民，X线胸片筛查疑似肺癌阳性604例，541例经过CT复查，诊断为肺癌363例（其中组织学确诊109例），CT复查校正筛查阳性率763.08/10万，世界人口标化率426.28/10万（95%CI：381.51/10万-471.05/10万), 男性482.78/10万，女性387.98/10万，男女比值1.24。各层肺癌流行强度差别较大，从A区→B区→C区→D区，肺癌筛查世标率逐渐减少，男女比值逐渐增大。筛查世标率最高的A区是最低D区的6.97倍。肺癌筛查阳性率与煤炭资源分布有关，调查对象肺癌筛查阳性率与烟煤使用率和使用量成正比，而与无烟煤使用率和使用量无明显关联。调查地区居民早期火塘使用率80%以上，其下无进气口，上无烟囱，煤炭燃烧不完全，加重煤烟废气污染。家族成员肺癌死亡率与肺癌筛查阳性率的分布一致，肺癌筛查阳性率随家族成员肺癌死亡率增加而增加。男性吸烟率85.08%，平均吸烟量16.12支/天；约50%从事过采煤、炼焦等工作。女性吸烟率1.37%，主要从事农业生产和做饭、喂养生猪等室内家务工作，吸烟、职业危害不是女性肺癌的主要发病因素，但可能是男性肺癌发病比女性严重的影响因素。

**结论:**

肺癌高发与室内外烟煤污染、家族易感性有关联，吸烟、采煤、炼焦不是女性肺癌的主要因素。

## Abstract (en)

**Background and objective:**

Xuanwei county is located at Late Permian coal-accumulating area in eastern Yunnan and western Guizhou, China. The lung cancer mortality rate in Xuanwei county is among the highest in China and has been attributed to exposure to indoor smoky coal emissions that contain very high levels of polycyclic aromatic hydrocarbons (PAHs). Recent years, the pollution and the higher mortality rate of lung cancer has been watched in the area around Xuanwei, and there is no report about whether the epidemic levels and the pathogen of lung cancer in other area of eastern Yunnan is similar to that in xuanwei. The aim of this study is to epidemic levels and cause of lung cancer in coal-producing area in the east of Yunnan province.

**Methods:**

382 study units (nature villages) were selected by stratified cluster random sampling from coalproducing area in eastern Yunnan province, China. The villagers who were aged 30-79 years with no history of lung cancer were enrolled. All the participants received an initial single-view posterior-anterior chest radiograph and administered a questionnaire survey (which involves the information of demography, household and fuel use, lifestyle, tobacco and occupational exposure history, family and personal medical history, etc. The subjects with a positive screen by chest x-ray underwent to have a computed tomography scan of the chest and biopsy examination. The confidence interval of the standardized rate ratio were adopted to evaluate the statistical significance of differences in different regions.

**Results:**

52, 833 villagers were surveyed and screened with X-ray. 604 of them were suspicious lung cancer with an initial chest radiograph, 541 underwent CT scan (362 were diagnosed by CT and 109 were diagnosed by histology). The adjusted positive rates for lung cancer screening with CT is 763.08 per 100, 000, the age-standardized rate (ASR) with the world standard population is 426.28 per 100, 000 (95% confidence interval=381.51/10 per 100, 000 to 471.05 per 100, 000), 482.78 per 100, 000 for man, 387.98 per 100, 000 for woman, male-to-female (M:F) rate ratios is 1.24. The intensity of lung cancer had significant difference between different study units. The ASR for lung cancer screening from A, B, C to D areas decreased in turn, and the area A was the highest of all, which was 6.97 times higher than the lowest area D, and the ratio between male and female was increased gradually. The positive rate for lung cancer was related to the distribution of coal and in direct proportion to the amount of smoky coal burning, but not associated with smokeless coal combustion. There are above 80% residents who burned " smoky" coal in indoor firepits which generated very high levels of air pollution. Lung cancer mortality of family members has the same distribution to positive rates of lung cancer screening, and they are in proportion to each other. 85% men smoke 16.12 cigarettes per day, averagely. About 50% of them did the job like coking, mining coal and so forth. Smoking rate of woman is 1.37%, they always did housework such as cooking, raising pigs and so on. Though the smoking and occupational hazard factors were not the major reasons for women to get lung cancer, they were possible reasons for men.

**Conclusion:**

In Xuanwei country of China, lung cancer was associated with exposure to smoky coal emissions and family susceptibility. Smoking, coking and mining were not the major risk factors lead to lung cancer for women.

滇东黔西晚二叠纪聚煤区是我国南方煤炭资源最为丰富的地区之一，不仅储量丰富、采掘历史悠久，而且煤炭品种齐全，分布着肥煤区、气煤区、焦煤区、瘦煤贫煤区、无烟煤区和褐煤区。20世纪70年代全国死因回顾调查^[1]^发现滇东宣威市肺癌疾患严重，农民肺癌死亡率居全国首位，女性肺癌死亡率是全国女性平均死亡率的8倍，男性是全国平均值的4倍。大量研究^[2]^表明宣威肺癌高发与室内烟煤燃烧产生的多环芳烃等污染物有关。近年来，宣威周边产煤地区(包括滇东、黔西)环境污染和肺癌高发问题日益突出^[3-5]^，近邻富源县2002年-2004年肺癌世界人口标化发病率达76.45/10万^[6]^，再次成为全国农村发病率最高地区之一。滇东产(燃)煤地区地理位置、自然环境、居民生活习俗和燃煤种类、方式与肺癌高发区宣威市基本一致，居民肺癌流行强度如何、致病因素是否与宣威有共同点等方面的问题一直是国内外关注的焦点。

为了全面了解产(燃)煤地区居民肺癌流行水平和发病原因，为预防控制肺癌提供科学依据，2007年由云南省政府专项基金资助，对滇东产(燃)煤区农民肺癌流行状况和发病因素进行抽样调查。

## 1 材料与方法

### 1.1 调查对象

根据滇东煤炭资源分布和肺癌历史发病、死亡资料采用多阶段、分层、整群抽样方法随机抽取样本，以样本地区30岁-79岁常住居民(不包括非农业、流动人口和既往恶性肿瘤病史者)为研究目标人群。

#### 1.1.1 样本量估计

由于肿瘤发生属于低概率事件，肿瘤的分布呈Poisson分布，样本量估计依据Poisson分布期望值可信限表^[7]^和滇东(产)燃煤区肺癌发病率的估计值(50/10万)推算^[2, 6]^。以往调查表明不同煤种、不同地区肺癌流行水平差别较大，按下式对每层样本量进行估计，总样本量约50, 000人。

$
n = \frac{4}{p} = \frac{4}{{\frac{{50}}{{100000}}}} = 8000
$

(*n*：层样本量；4：出现有效阳性病例的最低期望值；*P*：总体率估计。)

*N*=8, 000×4×1.5=48, 000≈50, 000 (*N*：总样本量)

#### 1.1.2 抽样

乡级样本：根据肺癌历史发病、死亡资料和滇东煤炭资源分布把曲靖市辖区内产(燃)煤乡镇分为4层：发病率或死亡率≥80/10万为高发区，次高发区50/10万-79/10万，中发区20/10万-49/10万，低发区 < 20/10万。无发病率或死亡率资料的乡镇以2003年-2006年当地县、市级主要医院新诊断的肺癌病例数为依据划分：高发区≥90例、次高发区60例-89例、中等发病区30例-59例和低发区 < 30例)。按比例(2:1)随机抽取23个乡级样本(其中高发区6个乡镇、次高发区8个乡镇，中发病区4个乡镇，低发区5个乡镇)。

村级样本：从每个乡级样本随机抽取2个-3个行政村，共抽取53个行政村。其中城乡结合区6个行政村，镇乡结合区7个行政区，农村村庄40个行政村。

调查小区(调查点)：每个村级样本随机抽取6个-8个居住人口比较集中的自然村作为整群抽样小区(调查点)，以满足每个乡级样本量2, 500-3, 000人的要求。被抽取的调查点30岁以上常住人口调查(问卷、X线胸片筛查等健康体检)覆盖85%以上。

### 1.2 调查方法

采用描述性流行病学研究方法和X线胸片肺癌筛查、痰癌细胞检测等方法对滇东产(燃)煤区肺癌流行状况和相关因素进行研究。

肺癌筛查：所有目标人群拍摄正侧位X线胸片，肺癌阳性胸片经临床诊断小组(3名以上县级医院放射医师)核实后，转诊CT复查，CT复查等阳性病例组织到上级医院进行细胞学、组织学诊断，其中咳嗽咳痰者现场采集痰样进行痰癌细胞分析。

随访观察：对CT复查诊断的阳性病例进行随访，跟踪观察对其生存状况进行评价。

流行病学问卷调查：调查对象问卷调查，内容包括人口学信息、吸烟、燃煤、炉灶使用、职业危险因素暴露、家族肺癌、慢性阻塞性肺气肿(chronic obstructive pulmonary disease, COPD)等疾病史等。

目标人群生活环境调查：近60年自然村周围5 km范围内煤炭、锌、铁冶炼、化工生产情况。

### 1.3 数据分析

所有数据录入Acces数据库，应用Excel 2007、SPSS 17.0进行数据分析。

#### 1.3.1 肺癌筛查阳性率(以下称肺癌筛阳率)

由于部分X线胸片筛查阳性疑似肺癌病例未复查CT，用年龄别CT复查阳性率(Rct)校正X线胸片筛查阳性疑似肺癌阳性率(Rx)。

$
肺癌筛查阳性率\left( {{{\rm{a}}_{\rm{i}}}} \right) = {{\rm{R}}_{\rm{X}}} \cdot {{\rm{R}}_{{\rm{CT}}}}
$

#### 1.3.2 标准化调整筛查阳性率(age-standardized screen positive rate)

以世界标准人口年龄构成标化调整，简称世标率(ASR)。

$
{\rm{ASR}} = \frac{{\sum\limits_{i - 1}^A {{a_i}{w_i}} }}{{\sum\limits_{i - 1}^A {{w_i}} }}
$

式中a_i_、w_i_分别为第i组年龄别肺癌筛查阳性率和标准人口的年龄组人口数；i为年龄组序号，i=1，2，3，……A。

#### 1.3.3 置信区间(confidence interval, CI)^[8]^

$
{\rm{ASR}} \pm {{\rm{Z}}_{{\rm{a/2}}}} \times \left[{{\rm{s}}{\rm{.e}}.\;\left( {{\rm{ASR}}} \right)} \right]
$

$
{\rm{Var}}\left( {{\rm{ASR}}} \right) = \frac{{\sum\limits_{i = 1}^A {\left[{{a_i}w_i^2\left( {100000-{a_i}} \right)/{n_i}} \right]} }}{{{{\left( {\sum\limits_{i = 1}^A {{w_i}} } \right)}^2}}}
$

$
{\rm{s}}{\rm{.e}}.\;\left( {{\rm{ASR}}} \right) = \sqrt {Var\left( {{ASR}} \right)} 
$

式中Z_a/2_——标准正态分位数，95%CI其取值1.96；*n*_i_——调查人群第i年龄组人口；Var(ASR)与s.e.(ASR)分别为ASR方差和标准差。

#### 1.3.4 标准化调整率的显著性检验

根据世界肿瘤研究中心(International Agency for Research on Cancer, IARC)推荐的方法，以标化率比(standardized rate ratio, SRR)的CI计算不同人群标化率间的统计学检验。如果95%CI包含1，则接受零假设，两者差别无统计学意义；如果95%CI不包含1，则拒绝零假设，两者差别有统计学意义^[8]^。

$
\begin{array}{l}
\;\;\;\;\;\;\;\;\;\;\;\;\;\;\;\;\;\;SRR = {\left( {\frac{{AS{R_1}}}{{AS{R_2}}}} \right)^{1 \pm {Z_{{\rm{a/2}}}}/X}}\\
其中{\rm{x}} = \frac{{AS{R_1} - AS{R_2}}}{{\sqrt {\left( {{\rm{s}}{\rm{.e}}.{{\left( {{\rm{AS}}{{\rm{R}}_1}} \right)}^2} + {\rm{s}}{\rm{.e}}.{{\left( {{\rm{AS}}{{\rm{R}}_2}} \right)}^2}} \right)} }}
\end{array}
$

## 2 结果

### 2.1 调查概况

共调查宣威、富源、师宗、麒麟、罗平5个产煤市、县、区的23个乡镇、53个行政村、382个自然村(表 1)。调查涉及乡级样本总体624, 910人(全年龄人口1, 507, 038人)。X线胸片筛查和流行病学问卷调查30岁-79岁52, 833人(全年龄人口134, 545人)，调查人数占乡级样本总体的8.36%，占乡级样本总体全年龄人口的3.47%。

**1:** 各县参与调查的乡、村、调查小区和调查人数 Asmple units and populations in different county

County	Villages and towns	Administrative villages	Natural villages	No. of participants			
				Male	Female	All	Sex rate
Fuyuan	8	18	144	9, 194	10, 678	19, 872	0.86
Xuanwei	10	25	200	10, 225	12, 292	22, 517	0.83
Qilin	2	5	18	2, 163	2, 654	4, 817	0.81
Shizong	1	3	10	1, 933	1, 978	3, 911	0.98
Luoping	2	2	10	842	874	1, 716	0.96
Total	23	53	382	24, 357	28, 476	52, 833	0.86

### 2.2 调查对象的依从性

由于样品量大、调查点多、农民外出务工(男性约20%，女性约8%)等因素影响，X线胸片筛查和问卷调查应答率70.69%(男性64.09%，女性77.53%)，与设计要求应答率85%以上有一定差距。参与X线胸片筛查和流行病学问卷调查的应答人群(依从者)、样本人群(包括依从者、非依从者)、调查涉及乡镇(乡镇样本2006年农业普查人口)男、女年龄结构分析，三组人群年龄结构无统计学差异(男性：χ^2^=7.478, 0，*P*=0.962, 9；女性：χ^2^=8.465, 8，*P*=0.933, 8)。应答人群男女比值0.86，明显小于样本人群及其涉及乡镇性别比值(二者男女比值分别为1.03和1.04)。

### 2.3 肺癌病例诊断

X线胸片筛查疑似肺癌阳性病例604例，其中541例(男277例，女264例)经过定点医院CT复查，CT检查诊断为肺癌363例。男、女CT复查率(CT复查数占X线胸片筛查疑似肺癌阳性病例的比例)分别为89.35%和89.75%(χ^2^=0.156, 8, *P*=0.692, 1)。男、女CT复查年龄分布见表 2，是否进行CT复查与其年龄分布无统计学联系，不同性别、年龄间CT复查率基本一致。

**2:** X线胸片筛查疑似病例CT复查情况 Diagnostic evaluation of positive lung cancer screen by X-ray with or without CT scan

Age (years)	Male			Female			All	
	CT scan	Unchecked CT^*^		CT scan	Unchecked CT		CT scan	Unchecked CT
30-	49	4		40	4		89	8
40-	58	5		55	8		113	13
50-	78	12		72	7		150	19
60-	67	10		67	7		134	17
70-	25	2		30	4		55	6
Total	277	33		264	30		541	63
*X*^2^	2.446, 0			0.778, 0			0.398, 7	
*P*	0.654, 3			0.944, 2			0.982, 6	
^*^chest X-ray examinations alone.								

调查地区医疗条件较差，肺癌诊断主要依靠X线胸部、CT和痰癌细胞检查，支气管纤维镜、淋巴结穿刺、经皮肺穿刺、胸腔镜、手术等细胞、组织学检查确诊病例较少。大部分年龄较低的CT诊断肺癌病例主动到省、市等上级医院进一步诊治，年老体弱病例由于经济条件限制和习俗等方面原因很少进一步诊治。随访搜集经省、市(曲靖)级医院住院病例和本地有关科研项目肺癌病例组织学、细胞学诊断资料，其中109例CT复查诊断的肺癌病例经过组织学、细胞学确诊，确诊率30.08%。男女性别、年龄组确诊率见表 3，低年龄组确诊率明显高于高年龄组，随着年龄增加，确诊率越来越低。

**3:** 各年龄组肺癌病例组组织、细胞学确诊率 No. lung cancers with CT scan diagnosed by biopsy by age

Age (years)	Male			Female			Female in NCI project^*^			All	
	*n*	No. (%) of biopsy examinations		*n*	No. (%) of biopsy examinations		*n*	No. (%) of biopsy examinations		*n*	No. (%) of biopsy examinations
30-	22	9 (40.72)		24	12(51.17)		11	5 (45.45)		46	21 (46.12)
40-	36	15(41.88)		43	16(36.83)		20	8 (40.00)		79	31 (39.11)
50-	52	12 (23.16)		58	18(31.08)		31	11 (35.48)		110	30 (27.34)
60-	50	8(16.15)		45	14(31.14)		28	14 (50.00)		95	22 (23.28)
70-	18	2(11.41)		15	3 (18.30)		7	3 (42.86)		33	5 (14.62)
Total	177	46 (26.02)		185	63 (33.95)		97	41 (42.27)		363	109(30.08)
^*^Collaborative Project between U.S. National Cancer Institute and China on"A Hospital-based Case-Control Study of Non-smoking Women in Xuanwei and Fuyuan".											

女性肺癌病例中97例被中美[美国国家癌症研究所(National Cancer Institute, NCI)]合作项目(简称NCI项目)——宣威、富源以医院为基础的女性肺癌病例对照研究项目征集为研究对象。项目病例除诊断方面给予经济资助、鼓励病例进行支气管纤维镜、经皮肺穿刺、手术等诊治外，还在痰癌细胞采集检测方法上加以改进。采用液基薄层细胞学技术^[9]^，采集病例首诊痰样、随后5日来晨痰和2月后晨痰，离心复集、用Thermo Shandon薄层细胞离心涂片机自动涂片16张以上，由痰检专家阅片，检查结果经中国医学科学院肿瘤医院专家复核，确诊率高达42.27%。

确诊病例中鳞癌占33.92%，腺癌占33.03%、细胞学检查未分类占16.60%，其它占17.43%。50岁前、后鳞癌、腺癌所占比例有明显差别(表 4)：50岁前腺癌占42.31%，鳞癌占25.00%；50岁后腺癌、鳞癌分别占24.56%和42.11%。

**4:** 确诊病例组织学分型 Histologic type distributions of lung cancers

Age (years)	Male						Female						Total				
	SCC	AC	BAC	SCLC	UDC		SCC	AC	BAC	SCLC	UDC		SCC	AC	BAC	SCLC	UDC
30-	7	9	2	2	4		6	13	3	4	2		13	22	5	6	6
50-	9	5	3	2	3		15	9	2	1	8		24	14	5	3	11
Total	16	14	5	4	7		21	22	5	5	10		37	36	10	9	17
%	34.78	30.43	10.87	8.70	15.22		33.33	34.92	7.94	7.94	15.87		33.94	33.03	9.17	8.26	15.6
AC: adenocarcinoma; SCC: squamous cell carcinoma; BAC: Bronchiolo-alveolar carcinoma; SCLC: Small cell carcinoma; UDC: Carcinoma, not otherwise specified.																	

### 2.4 病例转归

经过2年(2008年-2009年)的随访观察，CT检查阳性肺癌病例129例因肺癌死亡，男、女病死率分别为33.00%和38.29%(χ^2^=0.589, 78, *P*=0.442, 5)，病死率随年龄增大而增加。女性确诊肺癌病例与非确诊病例病死率基本一致(χ^2^=1.253, 7, *P*=0.262, 8)，但男性确诊病例病死率明显低于非确诊病例(χ^2^=7.754, 9, *P*=0.005, 3)。男性确诊病例中78.26%(36例)在省级医院治疗，45.66% (21例)经过手术切除术治疗；而女性确诊方式主要依靠痰检、纤支镜等方法确诊，只有部分家庭经济条件较好的病例或年轻病例到省级医院诊治，省级医院治疗占36.36%(23例)，手术治疗25.40%(16例)，大部分调查对象或仅进行化疗、放疗或基本未进行治疗(表 5)。

**5:** CT诊断肺癌病例和组织学确诊病例病死率 Age- and sex -specific fatality rate of lung cancer cases diagnosed by CT scan or histology

Age (years)	Male								Female						
	Cases diagnosed by CT scan only				Cases confirmed by biopsy				Cases diagnosed by CT scan only				Cases confirmed by biopsy		
	*n*	Death [*n* (%)]			*n*	Death [*n* (%)]			*n*	Death [*n* (%)]			*n*	Death [*n* (%)]	
30-	13	3	(23.27)		9	0	(0.00)		12	5	(39.64)		12	1	(8.33)
40-	21	7	(32.95)		15	2	(13.33)		27	8	(30.55)		15	4	(26.67)
50-	40	15	(38.27)		12	1	(8.33)		40	18	(43.91)		18	6	(33.33)
60-	42	19	(45.87)		8	1	(12.50)		31	15	(49.23)		15	7	(46.67)
70-	16	9	(58.89)		2	1	(50.00)		12	6	(48.96)		3	1	(33.33)
Total	132	53	(40.15)		46	5	(10.87)		122	52	(42.37)		63	19	(30.16)

NCI项目女性肺癌病死率42.27%，与全部女性肺癌病例病死率基本一致(χ^2^=2.142, 3, *P*=0.143, 3)，其中确诊病例病死率28.57%，非确诊病例病死率44.64%，两者之间无统计学差异(χ^2^= 0.306, 2, *P*=0.580, 0)(表 6)。

**6:** NCI项目*女性病例对照研究项目女性肺癌病例病死率 Fatality rate of lung cancer cases enrolled by NCI project^*^

Age (years)	Cases diagnosed by CT scan only			Cases confirmed by biopsy			Combined	
	*n*	Death [*n* (%)]		*n*	Death [*n* (%)]		*n*	Death [*n* (%)]
30-	6	2 (33.33)		5	0 (0.00)		11	2(18.18)
40-	12	5 (41.67)		8	2 (16.67)		20	7 (35.00)
50-	20	8 (40.00)		11	5 (25.00)		31	13 (41.94)
60-	14	8 (57.14)		14	8 (57.14)		28	16 (57.14)
70-	4	2 (50.00)		3	1 (25.00)		7	3 (42.86)
Total	56	25 (44.64)		41	16 (28.57)		97	41 (42.27)
^*^Collaborative Project between U.S. National Cancer Institute and China on"A Hospital-based Case-Control Study of Nonsmoking Women in Xuan Wei and Fu Yuan".								

### 2.5 肺癌筛查

#### 2.5.1 肺癌筛查阳性率

X线胸片筛查疑似肺癌阳性病例604例，其中541例进行CT复查(占89.72%)，CT复查诊断为肺癌363例，CT复查阳性率为66.91%，肺癌筛查阳性率为763.08/10万(男性917.53/ 10万，女性642.97/10万)，男女合计世标率为426.28/10万(95%CI: 381.51/10万-471.05/10万)，男性482.78/10万(95%CI: 415.08/10万-550.49 /10万)、女性387.98/10万(95%CI: 326.71/10万-449.24/10万)，男女世标率比值为1.24。

#### 2.5.2 不同层群肺癌筛查阳性率

根据设计要求，以乡级抽样单位为基础分为高发区Ⅰ(宣威市范围)、高发区Ⅱ(富源县、麒麟区范围)，次高发区Ⅰ(宣威市范围)和次高发区Ⅱ(富源、师宗县范围)、中发区、低发区(表 7)，各层肺癌筛查阳性率均有一定差别，从高发区→次高发区→中发区→低发区筛查阳性率逐渐减少。宣威来宾、西宁(高发区Ⅰ)肺癌筛查阳性率位居榜首，既明显高于次高发区、中、低发区，也高于高发区Ⅱ。通过SRR显著性检验，对无统计学差异的高发区、次高发区和中、低发区等设计分层进行合并调整为A区、B区、C区、D区(表 8)。

**7:** 设计分层的调查区域肺癌筛查阳性率（1/10万） Positive rate of lung cancer screening in designed investigating areas (per 100, 000)

Designed stratification	Sex	No. of participants	Positive rates for lung cancer screening	ASR (the 95% confidence interval)		s.e.(ASR)
The highest epidemic region Ⅰ (Laibin, Xining in Xuanwei county)	Male	3, 139	1, 770.83	887.36	(632.65-1, 142.07)	129.95
	Female	4, 120	1, 262.14	819.66	(549.23-1, 090.1)	137.98
	combined	7, 259	1, 485.57	828.04	(651.45-1, 004.63)	90.1
The highest epidemic region Ⅱ (Laibin, Housuo, Dahe, Mohong in Fuyuan county and Dongshan in Qilin county)	Male	4, 936	1, 041.51	645.62	(456.95-834.30)	96.26
	Female	5, 408	816.19	544.18	(356.16-732.20)	95.93
	combined	10, 344	924.52	592.24	(459.86-724.61)	67.54
The higher epidemic region Ⅰ (Longtan, longchang, Geyi, Tangtan in Xuanwei county)	Male	3, 823	982.07	491.06	(310.46-671.66)	92.14
	Female	5, 253	667.57	361.83	(200.41-523.25)	82.36
	combined	9, 076	799.92	422.98	(304.18-541.78)	60.61
The higher epidemic region Ⅱ (Zhuyuan, Zhongan, Yingshang in Fuyuan county, Xiongbi in Shizong county)	Male	4, 883	832.48	432.50	(290.74-574.25)	72.32
	Female	6, 488	489.28	278.76	(171.65-385.86)	54.65
	combined	11, 371	636.53	345.02	(258.54-431.5)	44.12
The medium epidemic region (Banqiao, Dongshan, Tianba, Yangchang in Xuanwei county)	Male	2, 398	302.56	176.89	(63.99-289.79)	57.6
	Female	3, 736	202.87	146.15	(49.32-242.99)	49.41
	combined	6, 134	241.95	159.24	(86.66-231.81)	37.03
The light epidemic region (Fucun, Laochang in Fuyuan county, Ciying in Qilin county, Agang, Majie in Luoping county)	Male	3, 928	163.93	105.51	(28.69-182.34)	39.2
	Female	4, 721	113.66	80.40	(11.31-149.49)	35.25
	combined	8, 649	136.49	91.34	(40.62-142.06)	25.88

**8:** 统计调整的调查区域肺癌筛查阳性率（1/10万） Positive rate of lung cancer screening in adjusted investigating area (per 100, 000)

Statistical adjusted investigating area	Sex	No. of participants	Positive rates	Age-standardized screen positive rate (the 95% confidence interval)		s.e.(ASR)
The region A (Laibin, Xining in Xuanwei county)	Male	3, 139	1, 787.85	887.36	(631.64-1, 142.07)	129.14
	Female	4, 120	1, 285.95	819.66	(547.54-1, 090.10)	136.14
	combined	7, 259	1, 506.8	828.04	(649.47-1, 004.63)	89.28
The region B (Housuo, Dahe, Mohong in Fuyuan county and Dongshan in Qilin)	Male	4, 936	1, 041.51	645.62	(456.95-834.30)	96.26
	Female	5, 408	816.19	544.18	(356.16-732.20)	95.93
	combined	10, 344	924.52	592.24	(459.86-724.61)	67.54
The region C (Longtan, Longchang, Geyi, Tangtan in Xuanwei county, Zhuyuan, Zhongan, Yingshang in Fuyuan county, Xiongbi in Shizong)	Male	8, 706	771.99	409.7	(304.62-514.78)	53.61
	Female	11, 741	503.11	284.94	(203.10-366.79)	41.76
	combined	20, 447	617.99	339.68	(274.30-405.05)	33.35
The region D (Banqiao, Dongshan, Tianba, Yangchang in Xuanwei county, Fucun, Laochang in Fuyuan county, Ciying in Qilin county, Agang, Majiein Luoping county)	Male	6, 326	255.6	141.93	(73.46-210.41)	34.94
	Female	8, 457	162.34	100.87	(44.20-157.54)	28.91
	combined	14, 783	202.25	118.74	(75.22-162.25)	22.2

A区、B区、C区、D区肺癌筛查阳性率、世标率差距较大，从A区→B区→C区→D区，肺癌筛查阳性率、世标率逐渐减少，世标率最高的A区是最低的D区的6.97倍, 而男女比值却逐渐增高，男女世标率比值依次为1.08、1.19、1.44、1.47。

#### 2.5.3 年龄别肺癌筛查阳性率

样本地区男、女性别肺癌年龄别筛查阳性率自30岁起随年龄的增大而同步增加(图 1)。男性高于女性，二者差别不大。45岁后，增加速度加快，且男女差距加大，男性筛阳率大大高于女性。但在65岁以后，男女筛阳率发生明显变化，女性反高于男性，至75岁高达3, 225.81/10万，比男性高20.51%。

**1:**
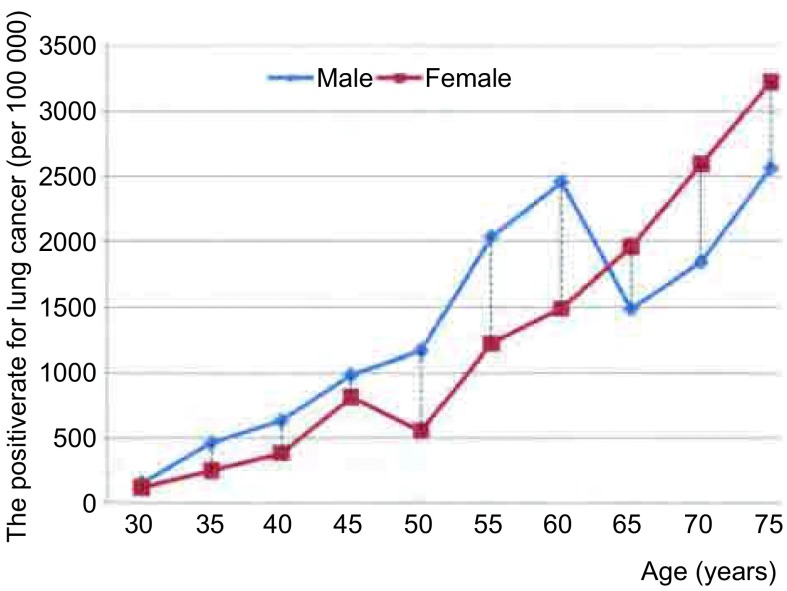
滇东产（燃）煤区年龄别肺癌筛查阳性率 Positive rate of lung cancer screening in coal-produing area in Eastern Yunnan by age and sex

图 2为调整分区肺癌年龄别筛查阳性率。A区、B区、C区、D区之间泾渭分明，A区、B区与C区、D区从30岁以后逐渐拉开距离，C区与D区之间也显示同样的趋势。B区与A区虽然有一定差距，但二者几乎平行上升。A区、B区男、女性别间年龄别筛阳率有所不同，60岁-前男性略高于女性，之后女性却明显增加，且高于男性。出现这种现象可能是偶合，也可能是其自身的规律。

**2:**
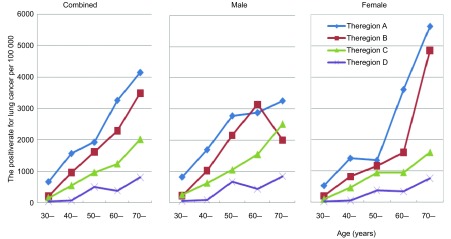
调整流调分区年龄别肺癌筛查阳性率比较 Positive rate of lung cancer screening in adjusted investigating area by age and sex

### 2.6 肺癌相关因素

#### 2.6.1 调查点煤炭生产、金属冶炼情况

表 9所示，62.62%调查点(自然村)周围5 km范围内生产烟煤，3.74%生产无烟煤，36.45%曾经有炼焦场(平均生产11.6年，最短生产2年，最长生产60年)，30.22%曾经进行铁、锌等金属冶炼(平均生产6.09年，最短生产1年，最长生产20年)，仅有8.7%有化肥厂(或化工厂)。大规模土法炼焦(煤炭焦化)和铁、锌冶炼主要集中在上世纪80年代和90年代，2000年后土法炼焦和铁、锌冶炼逐渐被取缔，除富源县大河、营上、竹园、中安和麒麟区东山、师宗县东山等少数乡镇仍有规模化炼焦厂外，其它地区基本停止炼焦和锌铁冶炼。

**9:** 各行政区域调查点（自然村）周围5公里近60年煤炭生产、金属冶炼情况 Coal-mine production and metal smelting conditions 5 km around sampling villages^*^ by county in recent 60 years

County	All Villages investigated†	Smoke coal producing			Smokeless coal producing			Coking			Metal smelting	
		Villages	(%)		Villages	(%)		Villages	(%)		Villages	(%)
Xuanwei	125	69	55.20		0	0.00		23	18.40		54	43.20
Fuyuan	143	103	72.03		10	6.99		67	46.85		26	18.18
Luoping	15	6	40.00		0	0.00		5	33.33		9	60.00
Qilin	18	11	61.11		0	0.00		11	61.11		5	27.78
Shizong	10	10	100.00		0	0.00		9	90.00		0	0.00
Total	311	199	63.99		10	3.22		115	36.98		94	30.23
^*^Study units; †Coal-mine production and Metal Smelting Conditions around the study units has been investigated actually.												

表 10为调整分区煤炭生产、金属冶炼情况。其中A区西宁街道办事处位于宣威市西北，无煤炭生产，但居民燃煤主要来源于近邻来宾镇。从A区(除其中西宁)到D区，生产烟煤自然村(调查点)所占的比例逐渐减少，而无烟煤生产仅限于D区；煤炭焦化也主要集中在A区、B区、C区，以主产焦煤和1/3焦煤的B区、C区为主；铁、锌冶炼分布没有明显趋势，主要集中在A区、D区，可能与当地生产的煤炭品种和质量有一定关系(A区、D区生产煤炭主要用作动力燃料，而不适于炼焦)。

**10:** 调整分区自然村周围5公里近60年煤炭生产、金属冶炼情况 Coal-mine production and metal smelting conditions 5 km around sampling villages in recent 60 years

Statistical adjusted investigating area	All villages investigated	Smoke coal producing			Smokeless coal producing			Coking			Metal smelting	
		Villages	(%)		Villages	(%)		Villages	(%)		Villages	(%)
The region A	22	13	59.09		0	0.00		9	40.91		13	59.09
The region B	55	55	100.00		0	0.00		29	52.73		12	21.82
The region C	101	85	84.16		0	0.00		58	57.43		22	21.78
The region D	133	46	34.59		10	7.52		19	14.29		47	35.34

#### 2.6.2 调查对象吸烟与职业

当地盛产优质烟草，居民吸烟率较高，而且一旦吸烟基本不间断，改变的只是烟草种类。经济条件好的购买价格高的卷烟，条件差的居民购买廉价卷烟或自制卷烟或抽吸烟丝。表 11所示，男性居民吸烟率85.05%，开始吸烟平均年龄20.53岁；其中92.74%主要抽吸卷烟，平均吸烟16.12支/天；7.26%主要用水烟筒和烟斗抽吸烟丝，平均抽吸223.72克/月。A区、B区、C区、D区各调整分区间吸烟率略有差异(χ^2^=14.642, 1, *P* = 0.002, 15)，但趋势不明显(线性趋势χ^2^=1.822, 3, *P*=0.177, 034)。女性居民吸烟率1.37%，开始吸烟平均年龄20.86岁；其中96.05%主要抽吸卷烟，平均日均吸烟15.46支；3.95%主要抽吸烟丝，平均每月抽吸223.61克。

**11:** 各调整分区吸烟状况、吸烟数量 Smoking status of participants in different adjusted region

Group	No. surveyed^*^	No. smoked (%)	Age starting to smoke (Mean±SD, years)	No. of cigarettes smoked on average per day (Mean±SD, cigarettes-d)
Male				
The region A	2, 436	2, 079 (85.34)	20.35±3.82	17.20±5.80
The region B	4, 707	3, 986 (84.68)	19.75±3.05	16.43±7.10
The region C	8, 508	7, 326 (86.11)	20.77±3.69	16.06±6.50
The region D	6, 287	5, 268 (83.79)	20.84±3.72	15.48±7.09
Total	21, 938	18, 659 (85.05)	20.53±3.62	16.12±6.75
Female				
The region A	3, 117	19(0.61)	21.11±3.09	15.59±6.09
The region B	5, 261	72(1.37)	20.25±3.83	15.00±4.95
The region C	11, 149	156(1.40)	21.16±5.02	16.30±6.31
The region D	8, 144	133(1.63)	20.85±3.32	14.70±6.69
Total	27, 671	380(1.37)	20.86±4.18	15.46±6.16
^*^Surveyed participants who respond these items of all questionnaire.				

调查对象主要从事农业生产，其中女性还承担生火做饭、煮猪食喂养生猪等室内家务工作，基本不从事采煤、炼焦、金属冶炼。村民依据当地其它行业的发展状况，男性短期从事有一定潜在健康危害的井下采煤、炼焦、金属冶炼等工作。表 12所示，49.24%的男性调查对象曾经从事过井下采煤工作，平均累计工作9.53年(2年-50年)；各调查调整分区井下采煤比例有明显差别(χ^2^=2, 146.425, 7, *P* < 0.001)，除A区外，B区到D区逐渐减少(线性趋势χ^2^=213.783, 4，*P* < 0.001)。6.63%的调查对象从事过炼焦工作，平均累计工作8.85年(1年-45年)，各调整分区炼焦比例有一定差别(χ^2^=755.223, 4, *P* < 0.001)，但趋势不明显(线性趋势χ^2^=2.726, 4，*P*=0.098, 7)，主产焦煤的C区、B区居民炼焦比例较高。2.25%的调查对象从事过金属冶炼工作，平均累计工作不到1年。

**12:** 调查对象从事掘煤、炼焦、金属冶炼等工作情况 Work other than farming of the study subjects during lifetime

Group	No. surveyed^*^	Coal mine				Coking				Metal smelting		
		*n*	%	Working years (Mean±SD, years)		*n*	%	Working years (Mean±SD, years)		*n*	%	Working years (Mean±SD, years)
Male												
The region A	2, 376	661	27.82	5.89±8.52		12	0.51	1.55±0.82		60	2.53	0.26±1.6
The region B	4, 584	3, 285	71.66	11.00±7.73		256	5.71	4.87±4.5		8	0.18	0.65±3.12
The region C	8, 399	4, 636	55.2	11.43±10.07		1, 009	12.2	10.6±8.78		213	2.66	0.48±1.67
The region D	6, 225	2, 045	32.85	6.09±7.94		142	2.27	3.64±3.86		191	3.10	0.65±2.69
Total	21, 584	10, 627	49.24	9.53±9.21		1, 419	6.63	8.85±8.28		472	2.25	0.52±2.22
Female												
The region A	3, 157	6	0.19	0.08±1.4		0	0	—		1	0.03	0±0.03
The region B	4, 757	54	1.14	4.90±7.63		8	0.16	2.14±1.21		3	0.06	0±0
The region C	11, 067	94	0.85	0.51±2.87		38	0.34	4.56±3.63		38	0.35	0.06±0.67
The region D	7, 886	37	0.47	0.08±1.04		8	0.10	2.8±1.79		7	0.09	0.01±0.33
Total	26, 867	191	0.71	0.31±2.24		54	0.20	4.02±3.34		49	0.18	0.02±0.43
^*^Surveyed participants who respond these items of all questionnaire.												

#### 2.6.3 生活燃料

调查对象主要使用烟煤作为生活燃料(表 13)，20岁前、后(女性婚前、婚后)烟煤使用率分别为84.59%和83.78%，平均使用量分别为2.83吨/年和2.65吨/年；从D区→C区→B区→A区烟煤使用率和年均使用量(燃煤量)逐渐增加[20岁前(女性婚前)线性趋势：χ^2^=1, 914.43，*P* < 0.001；使用量方差分析*F*=799.60，*P* < 0.001。20岁后(女性婚后)使用率：χ^2^=2, 109.36，*P* < 0.001；使用量方差分析*F*=278.54，*P* < 0.001]，A区烟煤使用率比D区高20%。无烟煤使用率较低，20岁前、后(女性婚前婚后)无烟煤使用率12.01%和11.32%，平均使用量分别为2.89吨/年和2.64吨/年。无烟煤作为生活燃料使用主要局限于无烟煤生产的C区、D区，其它调整分区较少使用无烟煤。

**13:** 居民20岁前后（女性结婚前后）生活燃料使用情况 Type of leading fuel used for cooking and heating at home in different times during lifetime

Group	No. surveyed^*^	Use of smoky coal			Use of smokeless coal			Use of coke	
		*n* (%)	Tons of fuel used per year (Mean土SD)		*n* (%)	Tons of fuel used per year (Mean±SD)		*n* (%)	Tons of fuel used per year (Mean ±SD)
Less than 20 years during lifetime									
The region A	7, 198	7, 008 (97.4)	3.19±1.19		118 (1.6)	2.12±1.56		72(1.00)	0.97±0.83
The region B	10, 281	9, 659 (94.0)	2.81±1.28		167(1.6)	2.39±1.43		455 (4.43)	1.12±0.63
The region C	20, 397	16, 567 (81.2)	2.77±1.25		3, 209 (15.7)	2.51±1.34		621 (3.45)	1.33±0.76
The region D	14, 780	11, 500 (77.8)	2.59±1.08		2, 828 (19.1)	3.46±1.42		452 (3.06)	1.03±0.77
Total	52, 656	44, 734 (85.0)	2.83±1.19		6, 322 (12.0)	2.89±1.46		1, 600 (3.04)	1.17±0.74
More than 20 years during lifetime									
The region A	7, 198	6, 985 (97.0)	3.01±1.23		104(1.4)	1.05±0.79		109 (1.51)	0.93±0.89
The region B	10, 281	9, 805 (95.4)	2.66±1.18		196(1.9)	1.09±0.8		280(2.71)	1.01±0.66
The region C	20, 397	15, 904 (78.0)	2.64±1.24		2, 829 (13.9)	2.39±1.51		1, 664 (8.16)	1.64±0.77
The region D	14, 780	11, 322 (76.6)	2.48±1.08		2, 877 (19.4)	3.39±1.58		581 (3.93)	1.00±0.76
Total	52, 656	44, 016 (83.6)	2.65±1.2		6, 006 (11.3)	2.64±1.65		2, 828 (5.36)	1.39±0.83
^*^Surveyed participants who respond these items of all questionnaire.									

#### 2.6.4 燃具炉灶

调查对象20岁前(女性婚前)日常生活使用的主要炉灶是火塘(在地上挖小坑、四周垒砖石而成，没有下进风口和烟囱)，使用率80.77%，主要设置在居民日常生活、休息的堂屋里，用于做饭、取暖、煮猪食、烘烤粮食等。地炉、高灶(设有下进风口和烟囱)使用率仅17.24%。各调整分区炉灶类型、放置位置、用途基本一致(表 14)。20岁后(女性婚后)日常生活使用的主要炉灶发生了显著变化，由原来单一的火塘发展为有烟囱灶、手提炉(室内、外移动)、火塘并举的多种形式，但不同肺癌筛查阳性率的地区未出现明显的趋势。

**14:** 居民使用的主要炉灶类型、放置位置和用途（%） The style, position and usage of stoves of villagers at home (%)

Group	Style of stove					Stove locations				Usuage of stove			
	Fire pit^*^	Open stove with chimney	Portable stove†	Others		Main hall	Kitchen	Outdoors		Cooking	Heating	Prepare food for pigs	Others
Less than 20 years during lifetime													
The region A	72.3	27.6	0.17	0.01		96.6	3.06	0.36		98.7	93.8	97.2	0.01
The region B	83.0	15.7	1.27	0.05		99.4	0.52	0.05		94.9	94.2	82.3	0.07
The region C	81.6	15.1	2.37	0.96		98.3	1.61	0.09		95.8	85.4	81.9	0.21
The region D	82.2	16.3	0.83	0.65		98.5	1.28	0.25		92.6	87.8	86.6	0.15
Total	80.8	17.2	1.42	0.57		98.3	1.5	0.17		95.1	88.9	85.4	0.14
More than 20 years during lifetime													
The region A	20.3	78.1	1.47	0.09		83	15.8	1.14		98.2	88.4	95.1	0.07
The region B	26.1	64.5	8.93	0.45		91.7	6.78	1.51		94.0	90.3	73.2	0.17
The region C	23.5	59.0	17.0	0.48		92.3	7.32	0.36		95.4	83.8	75.0	0.29
The region D	19.8	67.3	12.0	0.91		94.3	5.04	0.64		92.6	83.9	77.3	0.29
Total	22.8	64.7	11.9	0.54		91.5	7.73	0.77		94.7	85.7	78.0	0.24
^*^unvented stoves without chimneys; †Portable stoves are filled with coal and lighted once daily outdoors in the morning and brought indoors after visible smoke has diminished substantially and the coals are smouldering.													

#### 2.6.5 调查对象家族成员肺癌死亡率和COPD发病率

调查对象家族成员1980年后肺癌死亡与COPD、哮喘患病情况见表 15。调查对象父母肺癌死亡率、COPD、哮喘患病率明显大于其配偶、兄弟、姐妹；各调整分区家族成员肺癌死亡率从肺癌筛阳率较低的D区至筛阳率较高的C区、B区、A区逐渐增加(线性趋势χ^2^检验*P* < 0.000, 5)，A区各家族成员肺癌死亡率比D区高4倍以上。调查对象一级亲属(父母、兄弟、姐妹)肺癌死亡率(10.52‰)是配偶的1.67倍，肺癌筛查阳性率越高的地区二者的差距越大，且同辈男、女比值明显小于配偶男、女比值。各调整分区一级亲属、男性配偶COPD、哮喘患病率有一定差距(*P* < 0.001)，但仅有父、母、姐妹COPD、哮喘与肺癌筛查阳性率有关联(线性趋势χ^2^检验：父χ^2^=7.758, 7，*P*=0.005, 3；母χ^2^=54.82，*P* < 0.001；姐妹χ^2^=12.40，*P*=0.000, 4，兄弟、配偶COPD、哮喘患病率变化无明显趋势(*P* > 0.05)。

**15:** 调查对象直系亲属肺癌死亡率及COPD、哮喘发病率 Lung cancer mortality, incidence rate of COPD and Asthma of spouse and in immediate family members of the study objectives in eifferent epidemic areas of lung cancer, 1980-2006

Variable	The region A *n* (‰)	The region B *n* (‰)	The region C *n* (‰)	The region D *n* (‰)	Total
Husband					
No. of surveyed	2, 963	5, 160	10, 472	7, 864	26, 459
Lung cancer death	57 (19.24)	67(12.98)	86 (8.21)	38 (4.830)	248 (9.37)
Diagnosis of COPD	40 (13.50)	35 (6.78)	73 (6.97)	103(13.1)	251 (9.49)
Wife					
No. of surveyed	2, 195	4, 351	8, 133	6, 351	21, 030
Lung cancer death	16 (7.29)	16(3.68)	37 (4.55)	13 (2.05)	82 (3.90)
Diagnosis of COPD	8 (3.64)	15 (3.45)	30(3.69)	36 (5.67)	89 (4.23)
Brothers					
No. of surveyed	7, 899	14, 471	19, 654	17, 026	59, 050
Lung cancer death	124(15.70)	97 (6.70)	147 (7.48)	43 (2.53)	411 (6.96)
Diagnosis of COPD	51 (6.46)	57 (3.94)	77 (3.92)	96 (5.64)	281 (4.76)
Sisters					
No. of surveyed	6, 878	9, 995	22, 701	19, 020	58, 594
Lung cancer death	97 (14.10)	38 (3.80)	82 (3.61)	36(1.89)	253 (4.32)
Diagnosis of COPD	50 (7.27)	23 (2.30)	64 (2.82)	59(3.1)	196(3.35)
Father					
No. of surveyed	3, 797	7, 036	12, 883	9, 892	33, 608
Lung cancer death	236(62.15)	207 (29.42)	348 (27.01)	156(15.77)	947 (28.18)
Diagnosis of COPD	162 (42.67)	202 (28.71)	329 (25.54)	302 (30.53)	995 (29.61)
Mother					
No. of surveyed	3, 891	7, 186	6, 599	5, 845	23, 521
Lung cancer death	186(47.80)	100(13.92)	88 (13.34)	28 (4.79)	402 (17.09)
Diagnosis of COPD	159(40.86)	141 (19.62)	62 (9.40)	111 (18.99)	473(20.11)
^*^Chronic obstructive pulmonary disease had ever been diagnosed with chronic bronchitis and emphysema, 1980-2006.					

## 3 讨论

### 3.1 样本代表性与病例可靠性

本次调查采用多阶段、分层、整群概率抽样方法选取调查点，调查点分布基本与滇东煤炭资源分布一致，调查区域既覆盖肥煤、气煤、焦煤、无烟煤产区，又体现肺癌高、中、低发病区域的特点。由于样品量大、抽样点多、调查对象来源复杂、外出务工等对调查对象的依从性产生负面影响，应答率仅70.06%，未达到设计要求。其次X线胸片筛查阳性病例CT复查率89.72%，仍有10.28%X线胸片筛查阳性病例由于繁忙、交通不便、恐惧心理等影响未按设计要求进行CT复查。

无应答从性质上可分为有意无应答和无意无应答，前者常常与调查内容和方法有关，后者通常与调查内容本身无关，其出现是由其它原因造成的，如被调查者生病、外出、繁忙等原因不在现场，调查人员不熟悉地址无法或未找到被调查者。无意无应答可以看成随机的，虽然会造成估计量方差增大，但通常不会带来估计偏差^[10, 11]^。由于肺癌的发生主要与环境因素有关，其发病率、死亡率在人群、地域分布等方面差别较大，同一时段、同一地区则主要体现在性别、年龄差别方面。应答人群、样本人群与其代表的乡镇人群年龄结构一致，CT复查者和未复查者的性别、年龄结构无统计学差别，调查对象应答率和CT复查率对本次X线胸片筛查和问卷调查的影响不大。

细胞学、组织学诊断具有较高的可靠性，一直被作为肺癌确诊的国际金标准。调查地区医疗资源匮乏，当地居民经济条件较差，很少到外地进一步检查确诊。本次调查CT检查阳性病例中有109例被组织学、细胞学确诊，确诊率30.08%；其中97例女性肺癌病例被中美合作项目纳入研究对象，确诊率42.27%。低年龄组肺癌病例多选择到省级医院进一步治疗，其确诊率高、病死率低；高年龄组多选择就地治疗或放弃治疗，确诊率低、病死率高。男性未确诊病例病死率明显高于手术治疗比例较高的确诊病例，女性未确诊病例病死率与手术治疗比例较低的确诊病例病死率基本一致，从另一个侧面说明肺癌病例诊断的可靠性。

### 3.2 滇东产(燃)煤区肺癌流行病学特征

Martin M. Oken^[12]^报道美国PLCO项目对年龄为55岁-74岁67, 038名参与者进行胸部X线肺癌基线筛查，肺癌检出率1.9‰，其中现在吸烟者为6.3‰，过去吸烟者为4.9‰，从未吸烟者0.4‰。本次调查对产(燃)煤区居民52, 833人进行胸部X线片筛查，筛查对象年龄跨度(30岁-79岁)大于美国PLCO项目；CT复查校正肺癌筛查阳性率763.08/10万，世界人口标化率453.63/10万，高于PLCO项目高危人群。其次还呈现出以下特征：

男、女差别小，男性世标率482.78/10万(95%CI: 410.08-550.49)，女性世标率387.98/10万(95%CI: 326.71-449.24)，男女比值1.24。男女比值与肺癌筛阳率成反比，筛阳率最高的A区男、女筛阳率基本一致，男女比值1.03，比筛阳率最低D区小36.89%。

不同地区差别较大，A区世标率828.04/10万，比B区、C区分别高39.81%、143.77%，是D区的6.97倍。多次研究^[2, 5]^结果表明A区、B区一直是肺癌发病、死亡最严重的地区，其发病年龄比国内外发病率较高的美国、上海提前15年-25年。本次调查进一步表明产(燃)煤区发病年龄提前，A区、B区与C区、D区30岁-组后逐渐拉开差距，随年龄增大年龄别筛阳率差距越来越大。以往调查显示，宣威市、富源县年龄别发病率或死亡率至60岁后开始下降，而本次调查60岁后肺癌筛阳率仍不断上升，表明以往调查60岁以上老年人群由于经济、生活习惯等原因未能及时诊治而出现漏诊漏报，其肺癌发病率或死亡率可能被低估^[6]^。

### 3.3 产(燃)煤区肺癌相关因素

煤炭资源分布和使用(燃烧)与肺癌密切相关：滇东煤炭产量占全省的50%以上(烟煤占80%)，与黔西煤田构成长江以南最大的煤炭产区，区内煤炭资源丰富、品种繁多。本次调查点涉及肥煤、气煤、1/3焦煤、焦煤、贫煤、瘦煤、无烟煤产区和无煤区，调查人群既涵盖产煤区也包括非产煤区。调查点(自然村)周围烟煤生产比例较高的的A区、B区、C区肺癌筛阳率明显大于烟煤生产较少而无烟煤生产较多的D区。煤炭生产的分布决定着当地农村居民的使用，当地居民就近取煤作为生活、生产燃料。肺癌筛阳率与调查对象烟煤使用率和使用量成正比，而与无烟煤使用率和使用量没有明显关联，再次说明烟煤燃烧与肺癌高发有密切的关系。

本次调查地区居民燃煤炉灶、使用方式基本一致，早期(20岁前)火塘使用率80%以上。摆放在堂屋的火塘上无烟囱，无法排出煤烟等废气；下无进气口，氧气供给受限，煤燃烧不完全更进一步加重中间产物等废气的弥漫，对居民生活起居的室内造成严重污染，燃具炉灶使用和摆放位置对肺癌的发生起到重要作用^[13]^。

产(燃)煤区肺癌具有明显的家族聚集性：家族成员肺癌死亡率与肺癌筛查阳性率的分布一致，肺癌筛阳率随家族成员肺癌死亡率增加而增加。肺癌家族聚集性主要是家族成员长期生活在共同的环境中，与室内、外环境污染有关。不同肺癌流行强度的区域调查对象一级亲属肺癌死亡率大于配偶，肺癌严重的A区、B区一级亲属女性肺癌死亡率高于女性配偶，尤其是母亲肺癌死亡率比女性配偶高2.7倍以上。除环境、年龄因素外，家族成员易感性也起到重要作用^[14, 15]^。文献报道COPD与肺癌有关联^[16]^，本次调查家族成员除兄弟、配偶外，其它一级亲属COPD显示有一定关联。

吸烟、职业危害和工业污染不是(产)燃煤区肺癌的主要因素：吸烟是一般人群肺癌的主要因素之一，80%-90%的肺癌死亡归因为吸烟^[17]^。中国2002年吸烟调查显示^[18]^，国民男性吸烟率66.0%，女性3.08%；男性吸烟率最高的云南、青海等地吸烟率68.82%-80.6%，而且农村地区高于城市。调查对象男性吸烟率85.08%，女性基本不吸烟(1.37%)，吸烟率与全国调查结果一致。从A区→B区→C区→D区肺癌筛查阳性率差别较大，而吸烟率、开始吸烟年龄和吸烟数量基本一致。调查点工业基础薄弱，除煤炭生产外基本没有其它工业生产。女性除从事农业生产外，其它大部分时间在家里(室内)承担生火煮饭、煮猪食、喂养牲畜和照看老人、孩子等家务工作，暴露于室内煤烟污染的时间远远大于男性，女性肺癌筛阳率的高低与吸烟、职业危害、煤炭生产以外工业污染没有明显的关联。男性接近50%的调查对象从事过井下采煤、搬运等工作，其比例高低与肺癌筛阳率有一定关联。男性普遍吸烟，平均吸烟量16.12支/天，而且男性从事的掘煤、炼焦职业危害强而且缺乏自我保护工作比例大大高于女性。这些可能是男性肺癌发病、死亡比女性严重的影响因素。

何兴舟等^[2]^报道宣威肺癌高发区室内环境中BaP浓度与人群肺癌死亡率存在高度的相关性。Nakanishi等^[3]^报道云南富源县北部地区(烟煤产区)居民室内煤烟垢和烟煤燃烧产物有较高的致突变性，以烟煤为生活燃料的非吸烟男女性肺癌切除标本BaP浓度高达(608.7±477.1)pg/g，而日本非吸烟和重度吸烟肺癌切除标本仅为(180.1±104.5) pg/g和(207.5±98.8) pg/g。Lan^[19]^报道宣威地区发生肺癌危险性与生活用煤来源有关，其中肺癌最严重的来宾烟煤OR=24.8(95%CI: 12.4-49.6)，而羊场烟煤OR=3.9(95%CI: 1.8-8.3)。Tian等^[20]^研究发现宣威、富源肺癌高发还与脕二叠纪C1煤层的分布及高浓度纳米石英有关。这些研究说明宣威、富源等滇东产(燃)煤区不仅肺癌疾患严重，而且内部差异较大，致病因素复杂。本次研究样品量大、抽样点多、调查对象依从性、X线筛查灵敏度低等偏倚不可避免地影响研究结果。滇东乃至整个黔西产(燃)煤区肺癌流行水平、发病原因和发病机制尚待医学、地球物理化学、煤炭地质等多学科进一步研究。

## References

[1] Mumford JL, He X, Chapman RS (1987). Lung cancer and indoor air pollution in Xuanwei, China. Science.

[2] He XZ, Yang RD (1994). Lung cancer and indoor air pollution from coal burning.

[3] Nakanishi Y, Chen S, Inutsuka S (1997). Possible role of indoor environment and coal combustion emission in lung carcinogenesis in Fuyuan county, China. Neoplasma.

[b4] 4cdaTan XP. The fault of the environment. http://news.xinhuanet.com/zhengfu/2002-01/18/content_7220.24.htm, 2004-3-7.谭熙鹏. 都是环境惹的祸. http://news.xinhuanet.com/zhengfu/2002-01/18/content_722024.htm, 2004-3-7.

[b5] 5Zhou J, Gao S. The environmental costs of poverty. http://www.southcn.com/weekend/commend/20020815.htm, 2005-5-6.周浩, 高嵩. 脱贫付出的环境代价. http://www.southcn.com/weekend/commend/20020815.htm, 2005-5-6.

[6] Li JH, Zhang YS, Li Y (2008). An analysis of cancer incidence in Fuyuan, Yunnan province, during 2002-2004. J Environ Occup Med.

[7] Zhan SK (2003). Field survey techniques.

[8] Jensen OM, Storm HH, Maclennan R (1991). Cancer registration priciples and methods: Statistical methods for registries. Lyon: IARC Sci Publ.

[9] Liao ML (2008). Lung tumor.

[10] Jin YJ, Du ZF, Jiang Y (2008). Sampling techniques.

[11] Wang XT (2007). Is high response rate better? Another understanding on response rates for social survey. Sociological study.

[12] Oken MM, Marcus PM, Hu P (2005). Baseline chest radiograph for lung cancer detection in the randomized Prostate, Lung, Colorectal and Ovarian Cancer Screening Trial. JNCI J Natl Cancer Inst.

[13] Lan Q, Chapman RS, Schreinemachers DM (2002). Stove Improvement and risk of lung cancer in Xuanwei, China. J Natl Cancer Inst.

[14] Matakidou A, Eisen T, Houlston RS (2005). Systematic review of the relationship between family history and lung cancer risk. Br J Cancer.

[15] Jin YT, Xu YC, He XZ (2005). Familial aggregation of lung cancer in a high incidence area in China. Br J Cancer.

[16] Chapman RS, He X, Blair AE (2006). Improvement in household stovs and risk of chronic obstructive pulmonary disease in Xuanwei, China: retrospective cohort study. BMJ.

[b17] 17Smoking lung cancer-smoking lung cancer statistics, smoking lung cancer risk factors. http://www.lung-cancer-info-guide.com/smoking-and-lungcaner.html, 2006-10-10.

[18] Yang GH, Ma JM, Liu N (2005). Smoking and passive smoking in Chinese, 2002. Chin J Epidemiol.

[19] Lan Q, He X, Shen M (2008). Variation in lung cancer risk by smoky coal subtype in Xuanwei, China. Int J Cancer.

[20] Tian L, Lucas D, Fischer SL (2008). Particle and gas emissions from a simulated coal-burning household fire pit. Environ Sci Technol.

